# Intensity-modulated radiotherapy controls nasopharyngeal carcinoma distant metastasis and improves survival of patients

**DOI:** 10.1186/s40064-016-3117-1

**Published:** 2016-08-31

**Authors:** Xiaoqian Chen, Hao Lei, Zhongguo Liang, Ling Li, Song Qu, Xiaodong Zhu

**Affiliations:** Department of Radiation Oncology, Guangxi Medical University Cancer Hospital, No. 71 Hedi Road, Nanning, 530021 China

**Keywords:** Nasopharyngeal carcinoma, Metastasis, Intensity-modulated radiotherapy, Prognosis

## Abstract

**Background:**

This study evaluated the distant metastatic outcomes in nasopharyngeal carcinoma (NPC) patients treated with intensity-modulated radiotherapy (IMRT) plus chemotherapy.

**Methods:**

530 Non-metastatic NPC patients were retrospectively collected and reviewed after receiving IMRT with or without chemotherapy between June 2006 and December 2011. Patients were treated with one fraction of IMRT daily for 5 days a week for 69.96–74.09 Gy, while 473 (89.2 %) of patients also received chemotherapy.

**Results:**

Patients were followed up for a median follow-up duration of 49 months (range from 5 to 98 months). After treatment, 91 (17.3 %) patients developed distant metastasis. Distant metastasis after treatment was significantly associated with advanced 2010 Union for International Cancer Control (UICC)/American Joint Committee on Cancer (AJCC) T staging (*p* = 0.034), N stage (*p* < 0.001), 2010 UICC/AJCC stage (*p* < 0.001), and tumor recurrence (*p* = 0.029). However, chemotherapy failed to reduce cancer distant metastasis in early stage patients, the distant metastasis rate was 17.5 % in stage III and 24.2 % in stage IVA–B diseases, after IMRT and chemotherapy. The multivariate analysis showed that cancer remission duration, treatment modality, and metastatic site (*p* < 0.001, *p* = 0.027 and *p* = 0.022, respectively) were all independent predictors for overall survival of NPC patients after IMRT and chemotherapy.

**Conclusions:**

This study provided insight into the effects of IMRT plus chemotherapy in the treatment of NPC. Future studies will explore the efficacy of more aggressive systemic therapies for high-risk patients with distant metastasis.

## Background

Nasopharyngeal carcinoma (NPC) is one of the most common head and neck malignancies in Asia, in particular, in Southern China (Jia et al. 2006). Although the underlying mechanisms of NPC carcinogenesis have not been fully elucidated, current research has shown that a combination of multiple risk factors, such as viral infections, including Epstein-Barr infection, environmental risk factors such as consumption of salted fish, and genetic susceptibility may induce the development of NPC (Brennan 2006). NPC is more sensitive to radiotherapy and chemotherapy, compared with other cancers, which often leads to a favorable prognosis (Brennan 2006). In recent years, intensity-modulated radiotherapy (IMRT) as a novel radiotherapy technology was introduced into clinical practice. The treatment of patients with IMRT led to a significant improvement in the local recurrence-free survival and overall survival of NPC patients (Peng et al. 2012). Nevertheless, disease can progress despite treatment, in particular, the distant metastasis. Such complications therefore dramatically influence treatment efficacy and survival (Sun et al. 2014; Yue et al. 2014). To attain long-term remission of NPC, further studies are required in order to better understand the underlying molecular mechanisms of disease progression and identify clinical factors and treatment options.

Cancer metastasis is a multiple step process in which cancer cells migrate from the primary tumor site and enter into the lymphatic system, the bloodstream and/or directly invade neighboring tissues, and are then transported to a distant organ site and establish a secondary tumor lesion (Klein 2008). Multiple steps occur during metastasis, including angiogenesis, attachment of cancer cells to other cells and/or matrix proteins, translocation of neoplastic cells across the extracellular matrix barriers and proliferation at the secondary site (Woodhouse et al. 1997). Morphologically, cancer cells will undergo epithelial–mesenchymal transition (EMT) to increase their mobility and invasion capacity (Yang and Weinberg 2008) and then undergo metastasis. IMRT and chemotherapy induce apoptosis in cancer cells (Marin et al. 2015), thereby inhibiting cancer metastasis. However, during treatment, cancer cells can develop resistance to apoptosis inducing agents; thus, increasing the likelihood cancer recurrence may occur.

In this study, we retrospectively analyzed NPC patients after IMRT and chemotherapy. The association between clinicopathological features and cancer metastasis were evaluated. This study aimed to identify high risk factors for distant metastasis in NPC after IMRT and chemotherapy, furthermore, prospectively guide regarding treatment selection and duration for the patients.

## Methods

### Patients

In this study, we retrospectively collected 530 cases of NPC patients who received radical IMRT in Guangxi Medical University Cancer Hospital between June 2006 and December 2011. The inclusion criteria were (1) histologically confirmed NPC, (2) absence of metastasis, (3) absence of previous malignancy or other concomitant malignant disease, (4) no previous treatment, (5) Karnofsky performance status of 70 or more, (6) completion of radical radiotherapy during the study, and (7) absence of the development of metastasis during treatment. This study was approved by the Ethics Committee of Guangxi Medical University Cancer Hospital (Nanning, China).

### Radiotherapy and chemotherapy

Before IMRT, patients were underwent a contrast-enhanced CT to obtain IMRT specification. Briefly, patients were immobilized in the supine position with an individually manufactured precision mask from head to shoulders. After the target delineation finished, data were imported to the treatment planning system for treatment design. The prescribed radiation dose was 69.96–74.09 Gy to the planning target volume (PTV) of GTVnx (primary nasopharyngeal gross tumor volume) and GTVnd (involving cervical lymph nodes), 60–65.1 Gy to the PTV of CTV1 (high-risk regions), and 51.62–57.6 Gy to the PTV of CTV2 (low-risk regions and neck nodal regions). IMRT was delivered via nine fixed-gantry angles with step-and-shoot treatment techniques. All patients were treated with one fraction of IMRT daily, 5 days a week.

During the course of treatment, 473 (89.2 %) of these 530 patients also received chemotherapy (57 patients were alone treated with IMRT, in which 39 patients had early stage disease, another 18 patients with stage III-IVB disease did not receive chemotherapy due to physical, economic or personal reasons). Among these 473 patients, 122 patients were treated with concurrent chemotherapy (CCT) alone, 41 patients with neoadjuvant chemotherapy (NACT) followed by CCT, 251 patients with CCT plus adjuvant chemotherapy (AC), 37 patients with NACT + CCT + AC, and the remaining 22 patients received NACT alone or AC alone based on drug reactions or patient preference.

The regimens of NACT included two or three cycles of cisplatin 80 mg/m^2^ on day 1 and 5-fluorouracil 750 mg/m^2^/day by continuous intravenous infusion on 96 h every 3 weeks, or docetaxel 75 mg/m^2^ on day 1 and cisplatin 80 mg/m^2^ on day 1 every 3 weeks. CCT consisted of cisplatin 100 mg/m^2^ every 3 weeks or 40 mg/m^2^/week. For patients who received AC, cisplatin 80 mg/m^2^ on day 1 and 5-fluorouracil 750 mg/m^2^/day by continuous intravenous infusion on 96 h was repeated every 4 weeks for two or three cycles.

### Patient follow-up

After initial treatment, patients were followed up every 3 months during the first 2 years, every 6 months for the next 3 years, and then annually thereafter, unless a recurrence or metastasis was detected or the patient died. In an initial follow-up visit, patients were given a chest X-ray, abdomen ultrasound, magnetic resonance imaging (MRI) of the nasopharyngeal and cervical region, and laboratory tests. A CT scan of the chest and abdomen and a single-photon emission computed tomography (SPECT) scan of all bones were also performed every 6 months. If patients were suspected to have metastases, a CT and/or a SPECT scan of whole body were also performed to exclude cancer metastasis. Follow-up data were obtained from 98.5 % patients, with the median follow-up time being 49 months (range from 5 to 98 months). Distant metastases were classified as a single site in the bone, lungs, liver, or multiple sites of more than two organs.

### Statistical analysis

All statistical analyses were performed using the statistical package SPSS 16.0 (SPSS, Chicago, IL, USA). The overall survival time was calculated from the date of radiotherapy to the date of death or last follow-up. The χ^2^ test was used to assess association between cancer metastasis and clinical parameters. The Kaplan–Meier curves with a log-rank test were performed to calculate overall survival and distant metastasis rates. The Cox proportional hazard model was utilized for univariate and multivariate analyses of overall survival. All statistical tests were two-sided and a *p* < 0.05 was considered statistically significant.

## Results

### Characteristics of patients

In this study, we recruited 530 patients, including 394 males and 136 females (male to female ratio 2.9:1) with the median age of 44 years old (range between 16 and 79 years old). All patients were histologically diagnosed with NPC. To enroll into the IMRT protocol, all patients were given a complete physical examination, CT or MRI scan of the head and neck region, chest radiography/CT, abdominal ultrasound/CT, and whole body SPECT to exclude any distant metastases. Pathology classification of NPC was based on the World Health Organization (WHO) Grade I (squamous cell carcinoma), Grade II (non-keratinizing carcinoma) and Grade III (undifferentiated carcinoma) (Shanmugaratnam and Sobin 1993). Patients were staged according to 2010 Union for International Cancer Control (UICC) staging system (Edge 2010). Clinicopathological characteristics of these 530 patients are summarized in Table 1.

**Table 1:** Association of clinicopathological features with metastasis of NPC

Clinical characteristics	Metastasis		Total	*p* value
	Yes	No		
All cases	91	439	530	
Age (years)				0.250
≥44	52	222	274	
<44	39	217	256	
Gender				0.530
Male	70	324	394	
Female	21	115	136	
WHO histology				0.960
Grade I–II	46	223	269	
Grade III	45	216	261	
2010UICC/AJCC T staging				0.034*
T1	5	59	64	
T2	24	131	155	
T3	28	140	168	
T4	34	109	143	
2010UICC/AJCC N staging				<0.001*
N0	3	65	68	
N1	24	170	194	
N2	57	184	241	
N3	7	20	27	
2010UICC/AJCC staging				<0.001*
I	0	21	21	
II	9	105	114	
III	42	189	231	
IVA–B	40	124	164	
Treatment modality				0.075
Radiotherapy alone	5	52	57	
Chemoradiotherapy	86	387	473	
Recurrence				0.029*
Yes	15	39	54	
No	76	400	476	

*p<0.05

### Treatment complications

Acute treatment toxicities involved primarily reactions in the oral mucosa, salivary glands and leukocytes. The incidence rates of grade 1, 2 and 3 acute mucositis, xerostomia and leukopenia were 24.5, 45.1 and 30.4; 55.5, 39.4 and 0; 28.7, 60.2 and 4.7 %, respectively; however, no grade 4 toxicities observed (Table 2).

**Table 2:** Frequency of acute toxicity during IMRT

Toxicity	Grade				
	0 (%)	1 (%)	2 (%)	3 (%)	4 (%)
Mucositis	0	130 (24.5)	239 (45.1)	161 (30.4)	0
Xerostomia	27 (5.1)	294 (55.5)	209 (39.4)	0	0
Leukopenia	34 (6.4)	152 (28.7)	319 (60.2)	25 (4.7)	0

Late toxicities included xerostomia, hearing loss, and brain injury. Grade 1, 2 and 3 incidences were 68.9, 22.64 and 0.56 % for xerostomia; 3.02, 1.13 and 0.38 % for hearing loss; 5.1, 0.94 and 0.19 % for brain injury, respectively, while 2 patients developed grade 4 brain injury. Late radiation toxicity data are listed in Table 3.

**Table 3:** Late toxic effects of radiotherapy

Toxicity	Grade				
	0 (%)	1 (%)	2 (%)	3 (%)	4 (%)
Xerostomia	42 (7.9)	365 (68.9)	120 (22.64)	3 (0.56)	0
Hearing loss	506 (95.47)	16 (3.02)	6 (1.13)	2 (0.38)	0
Brain injury	495 (93.39)	27 (5.1)	5 (0.94)	1 (0.19)	2 (0.38)

### Effect of IMRT and chemotherapy on NPC patients

**Fig. 1:**
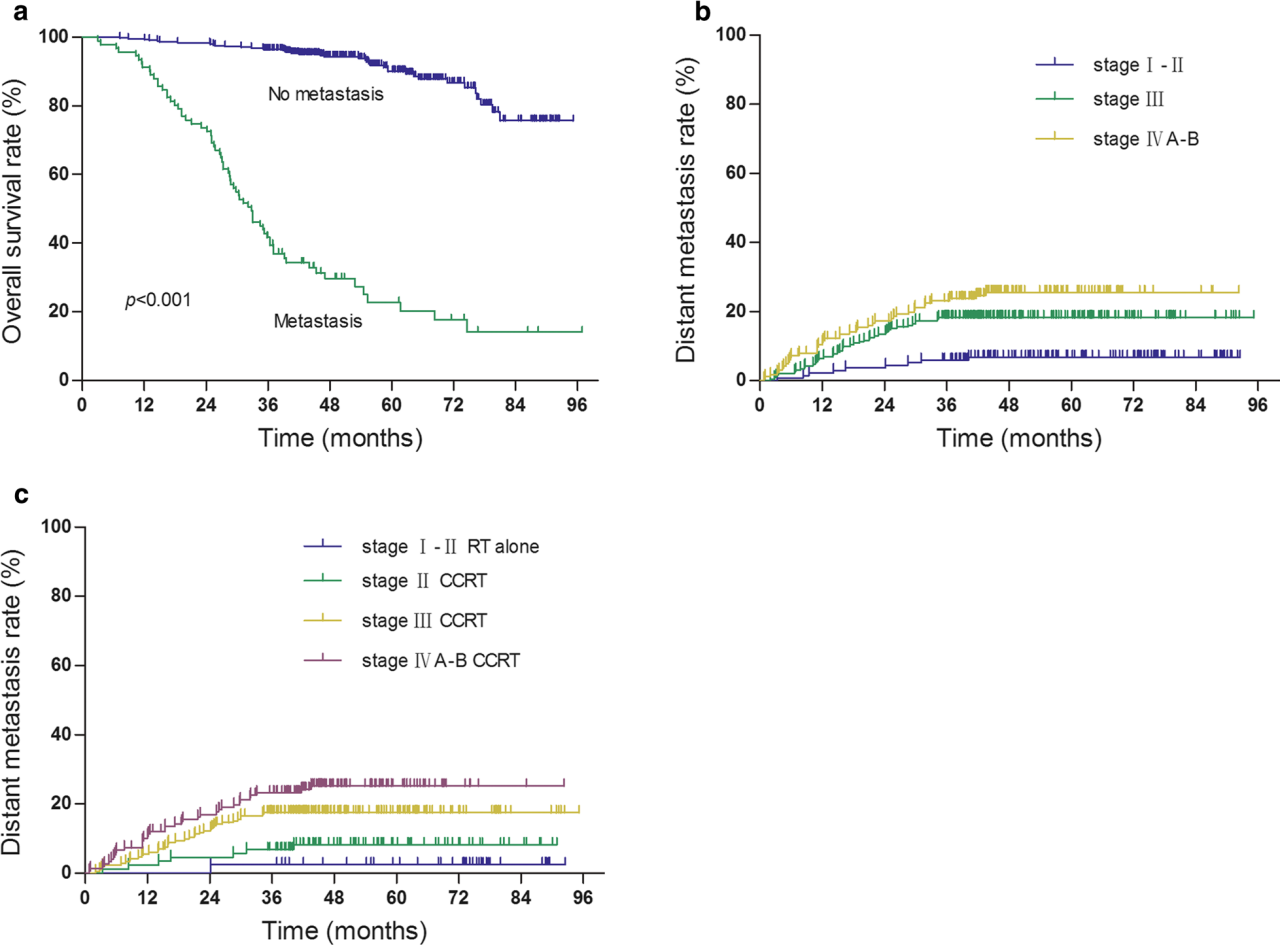
Kaplan-Meier curves of overall survival and distant metastasis rates. **a** Stratified by tumor metastasis. **b** Stratified by tumor staging. **c** Stratified by tumor staging with or without CCRT

At the end of this cohort study, 8 patients (1.5 %) were lost to follow-up, 101 (19.1 %) died during the study, 91 (17.2 %) developed distant metastases, and 54 (10.2 %) experienced local–regional failure. Statistical data showed that patients with metastases had a significantly poorer prognosis than those without metastases (Fig. 1a; Table 1), i.e., the 1, 3, and 5-year overall survival rates of patients with and without metastases were 91, 42, 29 and 99, 96, 93 %, respectively. Metastasis after IMRT was significantly associated with advanced 2010 UICC/AJCC T staging (*p* = 0.034), 2010 UICC/AJCC N staging (*p* < 0.001), 2010 UICC/AJCC stage (*p* < 0.001), and tumor recurrence (*p* = 0.029) (Table 1). Among the 91 patients with metastases, 7 also had local recurrence, 6 regional recurrence, and 2 local and regional recurrence.

With respect to cancer remission, we calculated duration from the end of IMRT to confirmation of metastasis. Treatment led to a median remission duration of 15 months (range from 1 to 44 months). The cumulative metastatic rates at 1-, 2-, and 3-years were 38.5 % (35/91), 70.3 % (64/91) and 95.6 % (87/91), respectively. Metastasis to the lungs was the most frequent, occurring in 43 patients, including single lung metastasis and combined with other organ metastasis, followed by bone metastasis in 38 patients and liver metastasis in 34 patients. The incidence rate of multi-organ metastasis occurred in 36.3 % (33/91) patients, while a single site of metastasis occurred in 23.1 % (21/91) in the lung, 19.8 % (18/91) in the bone, 14.3 % (13/91) in the liver, and 6.5 % (6/91) in other organs.

### Association of tumor staging with distant metastasis after treatment

Our data showed that the 5-year distant metastasis rate was 6.7 % in stage I–II, 18.2 % in stage III, and 24.4 % in stage IVA–B. Kaplan–Meier curve analysis revealed that survival of patients was significantly different between distant metastasis and tumor stages (χ^2^ = 17.401, *p* < 0.001; Fig. 1b). Distant metastasis rate was significantly lower in stage I–II than in stage III (χ^2^ = 9.490, *p* = 0.002) or stage IVA–B (χ^2^ = 17.983, *p* < 0.001), whereas there was no significant difference in distant metastasis rate between stage III and IVA–B (χ^2^ = 2.361, *p* = 0.105).

Furthermore, we assessed the effect of concurrent chemoradiotherapy (CCRT) on distant metastasis rate with different tumor staging after the removal of 18 stage III-IVB patients from data analysis due to a small sample size, and 22 patients who did not receive CCT in chemoradiotherapy group were also removed from this data analysis. We found that the corresponding 5-year distant metastasis rate was 2.6 % in stage I–II RT alone and 8 % in stage II CCRT (χ^2^ = 1.369, *p* = 0.242; Fig. 1c), whereas the 5-year distant metastasis rate was 17.5 % in stage III CCRT and 24.2 % in stage IVA–B CCRT. The distant metastasis rate was significantly lower in stage II CCRT patients than in stage III CCRT patients (χ^2^ = 4.411, *p* = 0.036; Fig. 1c) or in stage IVA–B CCRT patients (χ^2^ = 9.938, *p* = 0.002; Fig. 1c). However, there was no significant difference in the 5-year distant metastasis observed between stage III and stage IVA–B CCRT patients (χ^2^ = 2.772, *p* = 0.096; Fig. 1c).

### Univariate and multivariate analyses of prognostic factors in patients with tumor distant metastasis

**Fig. 2:**
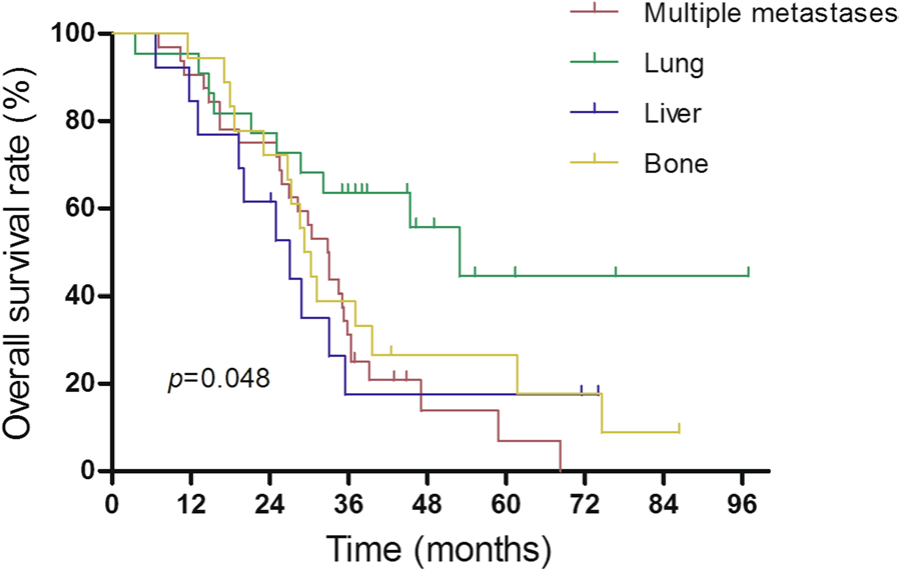
Kaplan-Meier curve of overall survival for patients with different metastatic sites

We then performed univariate and multivariate analyses to determine the factors that were associated with overall survival of patients with distant metastasis, using data of remission duration, age, gender, T stage, N stage, treatment modality, recurrence, and metastatic site (Table 4). Our univariate analysis showed that remission duration (HR 0.446; 95 % CI 0.276–0.721; *p* = 0.001), treatment modality (HR 0.303; 95 % CI 0.120–0.766; *p* = 0.012), and cancer metastatic site (HR 7.341; 95 % CI 1.610–33.479; *p* = 0.010) were significantly associated with overall survival in these patients, while the multivariate analysis further showed that these three factors were also independent predictors for overall survival of patients (*p* < 0.001, *p* = 0.027 and *p* = 0.022, respectively). Metastatic sites were mostly in the lung, bone, liver or multiple organs. Survival analysis showed that patients with lung metastasis alone had the best overall survival, followed by bone metastasis alone, liver metastasis alone and multiple metastases (*p* = 0.048, Fig. 2).

**Table 4:** Univariate and multivariate analyses of overall survival of patients with metastatic NPC

Variables	Univariate analysis			Multivariate analysis		
	HR	95 % CI	*p* value	HR	95 % CI	*p* value
Remission duration	0.446	0.276–0.721	0.001*	0.390	0.233–0.653	<0.001*
Age	1.513	0.925–2.474	0.099			
Gender	0.909	0.510–1.619	0.745			
2010UICC/AJCC T staging	1.134	0.875–1.469	0.341			
2010UICC/AJCC N staging	1.047	0.725–1.513	0.805			
2010UICC/AJCC stage	1.085	0.770–1.530	0.640			
Treatment modality	0.303	0.120–0.766	0.012*	0.344	0.133–0.885	0.027*
Recurrence	1.210	0.657–2.227	0.541			
Metastatic site	7.341	1.610–33.479	0.010*	5.972	1.292–27.609	0.022*

*p<0.05

## Discussion

Local recurrence and distant metastasis are the major causes of failure in treatment in NPC patients. In recent decades, 2- or 3-dimensional radiotherapy, in particular IMRT technology, has been used as the primary therapeutic approach to treat NPC patients and has shown remarkable benefits in local control and relapse-free survival of patients (Peng et al. 2012; Lai et al. 2011). However, distant metastasis is still the leading cause of IMRT treatment failure. To date, there were few studies that identify and evaluate the precise incidence of NPC distant metastasis after IMRT and chemotherapy. In the current study, we found that the distant metastasis rate in NPC patients was 17.2 %, which was similar to the results published in other IMRT studies (Sun et al. 2014; Yue et al. 2014; Lee et al. 2009; Leung et al. 2005). Additionally, we also found that the 1, 3 and 5-year overall survival rates of patients with and without distant metastasis were 91, 42, 29 and 99, 96, 93 %, respectively. Patients with distant metastases had a significantly poorer prognosis than patients without metastases. We also analyzed clinicopathological features as risk factors that contributed to NPC distant metastasis. Our results showed that advanced T and N stages, advanced clinical stages, and tumor recurrence were associated with the development of distant metastasis, while remission duration, treatment modality, and cancer metastatic site were all independent predictors for overall survival of metastatic patients after initial treatment. Our current data indicated that identification of patients with high risk to distant metastasis and choosing effective treatment regimes might lead to an increased rate of survival of patients.

In general, tumor T staging is related to local containment of the tumor, while the N staging is associated with distant metastasis (Leung et al. 2005; Li et al. 2014). However, our current data showed that compared to early T stage patients, advanced T stage patients had a higher distant metastasis rate. In stage T3–4 patients, destruction of the base of skull was a common finding. A previous study Cheng et al. (2005) reported that NPC commonly invades into the parapharyngeal venous plexus along with the marrow of the skull base. Although IMRT improves the target dose to the nasopharyngeal region and protects tissues near the skull base such as the brain stem and temporal lobe, some targeting doses may not be sufficient to eliminate tumor lesions, resulting in an increase in distant metastasis. Furthermore, a previous study demonstrated that tumor N staging was a predicative factor for distant metastasis (Xia et al. 2013). Our current data were consistent with previous findings. Other studies have also revealed that distant metastasis-related free survival was proportional to the N staging of the disease (Sun et al. 2014; Wang et al. 2014; Wong et al. 2010). Although IMRT provides excellent regional treatment of NPC, distant metastasis-related free survival was still low in patients with advanced N stage disease (Peng et al. 2012). This may be due to a higher micrometastatic risk in such patients and an increased IMRT dose may be unable to control such micrometastatic lesions (Sun et al. 2014).

Furthermore, our current study showed that the local recurrence rate was 10.2 % (54/530). Specifically, 15 of 54 patients presented with distant metastasis during follow-up. Two previous studies have shown that patients with regional recurrence had an increased risk of developing distant metastasis, and thus, regional recurrence has been recognized as an independent risk predictor for distant metastasis in NPC patients (Peng et al. 2013; Kwong et al. 1994). In accordance with these studies, our current data also showed a significant association between tumor recurrence and distant metastasis. Additionally, remission duration after treatment was an important prognostic factor for overall survival of patients with metastatic NPC (Teo et al. 1996). Our current data demonstrated that the median remission duration in patients was 15 months, which was similar to the median remission of 13 months in another large cohort of NPC patients treated with 2-dimensional radiotherapy (Yi et al. 2006). Our current data also showed that most distant metastasis occurred within 3 years following initial treatment, with the cumulative metastatic rate of 95.6 % (87/91). These data indicate that it may be necessary to closely monitor for distant metastasis within 3 years after treatment. In addition, it is paramount to choose an appropriate treatment regime in NPC patients, particularly those at high risk of metastases.

In our current study, most patients received CCRT based on their clinical staging. The 5-year distant metastasis rate in early stage disease was low, which did not associate with IMRT alone or CCRT. To date, there is still no consensus that whether stage IIb patients require chemotherapy. A previous study reported that IMRT combined with CCT did not show any benefit in patients with stage IIb NPC (Tham et al. 2010). As recommended by National Comprehensive Cancer Network (NCCN) clinical practice guidelines of head and neck cancers, concurrent cisplatin-based chemoradiotherapy is the standard treatment for locally advanced NPC. Our current study showed that CCRT treatment of patients with stage III and stage IVA–B diseases had 17.5 and 24.2 % of the 5-year distant metastasis rate, respectively. For locally advanced patients, treatment outcome was not satisfactory even with aggressive treatment regimes (Li et al. 2015). CCRT alone or CCRT in combination with other chemotherapeutic agents in locally advanced NPC patients showed no difference in overall survival and distant metastasis-free survival, only NACT + CCRT improved the distant metastasis-free survival compared to RT alone (Sun et al. 2013). Theoretically, NACT could improve survival by reducing tumor burden and targeting micrometastasis. However, additional studies Wu et al. (2014), Qiu et al. (2016) have not supported these findings. One possible reason for the conflicting results in these studies may be differences in patient inclusion criteria. Thus, identifying high-risk metastatic NPC patients and applying novel chemotherapeutic regimens including molecular targeting agents to treat such patients should be the focus of future studies.

In addition, our current data showed that the most common sites of cancer metastasis was the lung, followed by the bone and liver, along with multi-organ metastasis, which was consistent with previous studies (Yi et al. 2006; Hui et al. 2004; Cao et al. 2013). The metastatic timing and sites after both IMRT and 2-dimensional radiotherapy were similar, indicating that IMRT had a limited effect on the control of NPC distant metastasis (Zhang et al. 2015). Previous studies have shown that patients with lung metastasis alone had better survival (Hui et al. 2004; Cao et al. 2011), whereas prognosis of patients with liver metastases had a lower survival rate, and those with bone metastasis had a lower survival rate, although higher than patients with liver metastases (Tian et al. 2013). Similar results were observed in our current study. Compared to 2-dimensional radiotherapy, the survival benefits of IMRT possibly originated from high rates of locoregional control. Thus, novel treatment strategies are needed in order to reduce distant metastasis. Furthermore, data from the literature has indicated that age, remission duration, and tumor T stage were significant predictors for survival of metastatic NPC patients (Teo et al. 1996). Our current study only showed that remission duration, treatment modality, and metastatic site were independent predictors for overall survival of metastatic patients after IMRT and chemotherapy. Most patients with cancer distant metastasis were locally advanced (86/91 cases) and compared to IMRT alone, chemoradiotherapy could improve the prognosis of this group of patients.

## Conclusion

This study assessed the effects of IMRT and chemotherapy on NPC patients without initial distant metastasis and showed that advanced T, N, and clinical stages plus tumor recurrence all contributed to the development of distant metastasis. IMRT had no impact on metastatic timing and sites of distant metastasis, and remission duration, treatment modality, and metastatic sites were all independent predictors for overall survival of metastatic NPC patients after IMRT and chemotherapy. Thus, more effective treatment regimes should be explored to manage patients with high distant metastasis risk in the future.

## Abbreviations

NPC: nasopharyngeal carcinoma
IMRT: intensity-modulated radiotherapy
EMT: epithelial–mesenchymal transition
CCT: concurrent chemotherapy
NACT: neoadjuvant chemotherapy
AC: adjuvant chemotherapy
MRI: magnetic resonance imaging
SPECT: single-photon emission computed tomography
WHO: World Health Organization
UICC: Union for International Cancer Control
NCCN: National Comprehensive Cancer Network
CCRT: concurrent chemoradiotherapy
